# Bilateral Simultaneous Heterotopic Ossification of the Reflected Head of Rectus Femoris Muscle: A Case Report and Review of the Literature

**DOI:** 10.1155/2014/497075

**Published:** 2014-04-03

**Authors:** Murat Tonbul, Seyma Ozen, Ayse Tuba Tonbul

**Affiliations:** ^1^Department of Orthopedics and Traumatology, Private Reyap Hospital, Turkey; ^2^Ozel Reyap Hastanesi, Ortopedi ve Travmatoloji Klinigi, Omurtak Cad. No. 48, Corlu, Tekirdag, Turkey; ^3^Department of Physical Therapy and Rehabilitation, Private Reyap Hospital, Turkey; ^4^Department of Nuclear Medicine, Corlu State Hospital, Tekirdag, Turkey

## Abstract

Lamellar bone formation in an abnormal location is defined as heterotopic ossification. It commonly occurs around the hip joint and most often involves the abductor muscles. It is a benign condition; however, its etiology remains largely unknown. Most previously reported cases have been due to trauma or intramuscular hemorrhage. In this paper, we present a case of bilateral heterotopic ossification of the reflected head of rectus femoris muscle without antecedent trauma or any other known cause, as the first and unique case in the literature. She was treated by excision of the right symptomatic bony mass via a modified Smith-Petersen approach. Postoperatively, she received 75 mg indomethacin daily for six weeks. She was pain-free and obtained full range of motion 3 weeks after the first intervention.

## 1. Introduction

Lamellar bone formation in an abnormal location is defined as heterotopic ossification. It is first described by Patin in 1962 [1]. It is a benign condition; however, its etiology remains largely unknown. The most common cause is trauma [2]. Although it is a self-limiting condition, it can cause significant morbidity, as restriction of range of motion, when it occurs close to joints [2, 3].

Heterotopic ossification commonly occurs around the hip joint, especially after total hip arthroplasties, and most often involves the abductor muscles [4, 5]. Involvement of the iliopsoas and quadratus muscles, as in very few cases, has been reported [1, 6–9]. Most previously reported cases have been due to trauma or intramuscular hemorrhage. In this paper, we present a case of bilateral heterotopic ossification of the reflected head of rectus femoris muscle without antecedent trauma or any other known cause, as the first and unique case in the literature.

## 2. Case Report

A 35-year-old amateur dancer woman presented to our outpatient department with a history of gradual, progressive, and painless restriction of movement of her right hip over the previous five years. There was no history of surgery, bleeding disorder, or systemic illness.

**Figure 1:**
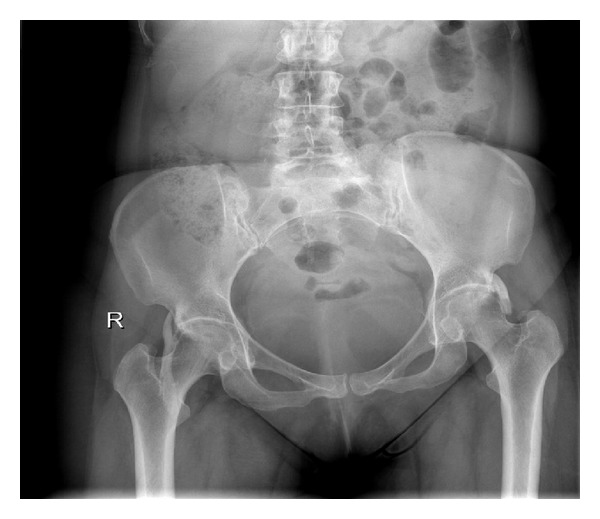
Anteroposterior X-ray of the pelvis at the initial presentation.

**Figure 2:**
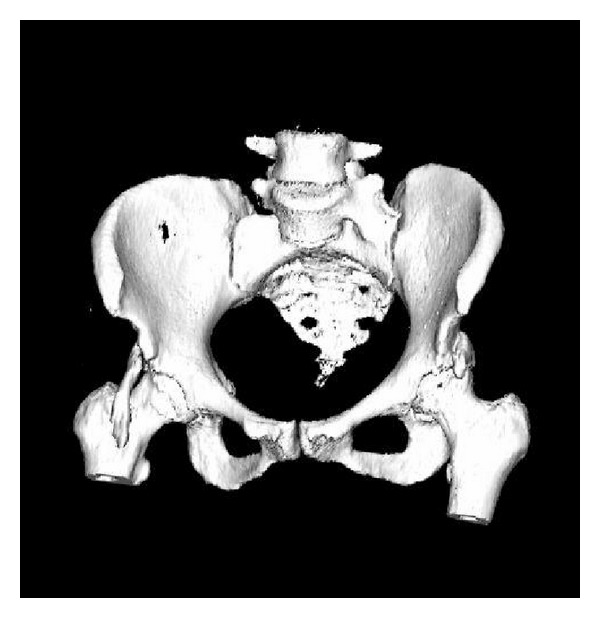
3D-BT of the pelvis, presenting bilateral heterotopic ossification masses.

Local clinical examination revealed fullness in the right femoral triangle, but any palpable mass. There was no distal neurovascular deficit. She had significant restriction of hip flexion and internal rotation. Wasting in her hip and thigh musculature was absent. Radiological evaluation revealed bilateral ossified mass lying anterior to the hip joint originating from the anterior superior margin of the acetabulum and extending to the lesser trochanter (Figure 1).   A 3-dimensional computerized tomography (3D-CT) scan revealed bilateral well-defined masses (Figure 2). The ossified mass measured approximately 10 cm at right and 5 cm at left. A three-phased nuclear scan is obtained in order to define its maturity.

We discussed the treatment options with the patient after making a diagnosis of heterotopic ossification, explaining the need for surgical excision for the symptomatic right hip and the appropriate timing of surgery. We gave detailed information about the prophylactic postoperative process, including the need of single dose of 700 cGy radiotherapy and indomethacin medication 75 mg orally for six weeks.

**Figure 3:**
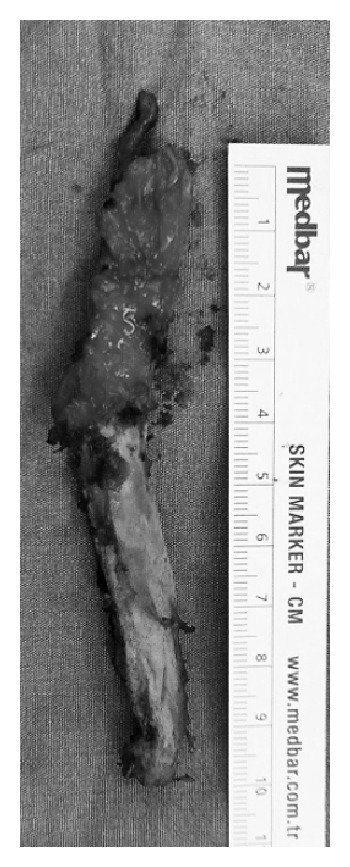
Photo of the excised mass.

The patient was placed in a supine position with a sand bag under the ipsilateral hip. The skin was incised starting from the anterior superior iliac spine and curving down so that it runs vertically for 8 cm, heading toward the lateral side of the patella (modified Smith-Petersen incision). The incision was deepened first between the sartorius and the tensor fasciae latae and then between the rectus femoris and the gluteus medius. We could then find the 10 cm long bony mass and carefully isolated it with minimal bleeding, osteotomized at the proximal end, stripped from the adherent soft tissue, and removed (Figure 3). Intraoperatively, we confirmed a full passive range of movement, achieved local homeostasis, and closed the incision over a suction drain. Because she rejected having even a single dose of 700 cGy radiotherapy, indomethacin medication 75 mg orally for six weeks was started postoperatively. Histopathological examination of the excised mass confirmed the diagnosis of myositis ossificans. Postoperative radiographs confirmed that the entire mass has been excised. The patient started a regimen of active assisted range of hip movements from the second postoperative day. She was able to sit cross-legged and even squat by the fifth postoperative day; she retained the range of motion achieved at surgery till her last followup at 24 months.

## 3. Discussion

The abductor musculature is the most common site where heterotopic ossification around the hip is observed. The iliopsoas and the quadratus muscles are rarely reported as the involvement sites [1–3, 6–9]. Although it may have been very rarely reported, to our knowledge and review of the literature, we could not find any report about the reflected head of the rectus femoris to be involved as a region of heterotopic ossification.

The pathogenesis of heterotopic ossification is still elusive. Trauma is reported to be the most common cause; however, atraumatic causes have also been reported [9]. Although no distinct trauma story has been mentioned by the patient, we suggest that repetitive microtrauma caused by dancing may have been the etiology of our case.

The principal disability in nonprogressive heterotopic ossification is usually mechanical and characterized by painless restriction of joint movements, the degree of limitation being dependent upon the amount, and site of ossification. Patients usually regain a full active range of motion within a few days of excision of the bony mass [2, 3].

Direct radiographs, CT scans, MRI, ultrasound imaging, and three-phase scintigraphy all play distinguishable roles in monitoring the disease [9–12]. She initially presented us with her direct radiograph and MRI of her pelvis. We opted her conventional and 3D-CT scans for the exact extension of the lesions and three-phase bone scan for their maturity.

Preventive measures, such as meticulous dissection during surgery, appropriate immobilization and physical therapy, indomethacin medication, and prophylactic radiation therapy, are the mainstay of the management of heterotopic ossification [4, 5, 13, 14]. The principal of the treatment is appropriate immobilization for immature bone and physical therapy for a mature mass. Bisphosphonates and radiation therapy have been used for treatment, but their efficacy is questionable especially in spinal cord injuries [14]. In patients with restricted joint movements, because of heterotopic ossification mass, the choice of treatment is surgery. The timing of surgical excision of the mass, intraoperative dissection, and taking care to minimize the recurrence all determine the outcome. Dissection in the intermuscular planes, minimal periosteal stripping, and achieving good homeostasis before closure are all technical tips for reducing the recurrence. Postoperative low dose radiotherapy and indomethacin medication are the other measures that may reduce the recurrence.

A very important issue, especially in nontraumatic cases, is the differential diagnosis, which includes malignant conditions like osteosarcoma and chondrosarcoma, infections, calcified abscesses, and hematomas [9]. However, a well-defined trabeculation pattern and mature peripheral lamellar bone formation, diagnosed by means of direct X-rays and CT scans, should clarify the diagnosis and prevent the patient for further unnecessary biopsies and premature surgical intervention.

Taking in mind that heterotopic ossification can develop without antecedent trauma, excision after the maturation of the bony mass, taking care for surgical skills, and postoperative prophylactic measures, such as indomethacin medication and radiotherapy, ensure a good result for treating this chemical-mechanical block to motion.
